# Image-Processing-Driven Modeling and Reconstruction of Traditional Patterns via Dual-Channel Detection and B-Spline Analysis

**DOI:** 10.3390/jimaging11100349

**Published:** 2025-10-07

**Authors:** Xuemei He, Siyi Chen, Yin Kuang, Xinyue Yang

**Affiliations:** School of Design and Art, Shaanxi University of Science and Technology, Xi’an 710021, China; yuec2798@gmail.com (S.C.); kuangyin629@163.com (Y.K.); syfzyxy1@163.com (X.Y.)

**Keywords:** image processing, dual-channel, pattern reconstruction, graphic primitives, B-spline curves, fractal analysis

## Abstract

This study aims to address the research gap in the digital analysis of traditional patterns by proposing an image-processing-driven parametric modeling method that combines graphic primitive function modeling with topological reconstruction. The image is processed using a dual-channel image processing algorithm (Canny edge detection and grayscale mapping) to extract and vectorize graphic primitives. These primitives are uniformly represented using B-spline curves, with variations generated through parametric control. A topological reconstruction approach is introduced, incorporating mapped geometric parameters, topological combination rules, and geometric adjustments to output topological configurations. The generated patterns are evaluated using fractal dimension analysis for complexity quantification and applied in cultural heritage imaging practice. The proposed image processing pipeline enables flexible parametric control and continuous structural integration of the graphic primitives and demonstrates high reproducibility and expandability. This study establishes a novel computational framework for traditional patterns, offering a replicable technical pathway that integrates image processing, parametric modeling, and topological reconstruction for digital expression, stylistic innovation, and heritage conservation.

## 1. Introduction

The advancement of digital technologies has provided new technical platforms for the protection, inheritance, and innovation of traditional culture [1]. Traditional crafts and intangible cultural heritage have a long history, within which richly shaped traditional patterns, serving as visual symbols of material culture, constitute a key element of cultural esthetic systems. With the development of digital technology, computer-aided design has been widely applied in architecture, industrial design, and related fields, and research on traditional patterns has increasingly undergone digital transformation [2,3].

Academics have made a series of achievements in the digital expression and cultural deconstruction of traditional patterns. For instance, Zhang disassembled the Bada halo pattern into graphic primitives such as filling, skeleton, and decoration, and reconstructed its structural model in a parametric way, ultimately realizing controllable derivation of pattern styles and adaptive color filling [4]. Wen analyzed the morphological spectrum of enamelware patterns via cultural gene theory, and deduced innovative styles based on shape grammar that were applied to tea packaging design [5]. Yang analyzed the patterns of opera costumes using image recognition and digital analysis techniques and quantitatively categorized these patterns, further developing a design platform with style learning and re-generation capabilities that ultimately provided a paradigm reference for the digital design of traditional patterns [6].

From the perspective of existing research, although many methods achieve high accuracy in shape reconstruction and style extraction, problems persist, including unclear structural expression and fragmented generation mechanisms. On the one hand, modeling approaches based on image recognition are often overly dependent on visual features and lack a systematic expression of the structural hierarchy of patterns. On the other hand, traditional vision-driven methods lack an extensible graphic primitive modeling framework, making it difficult to combine and parameterize patterns under a unified logic. The main contributions of this study are threefold: (1) a dual-channel image processing pipeline for robust primitive extraction; (2) a unified B-spline-based parametric modeling framework for graphic primitives; and (3) a topological reconstruction method enabling systematic pattern variation and complexity quantification [7,8,9,10,11].

## 2. Materials and Methods

### 2.1. Research Materials

Traditional patterns constitute a fundamental component of cultural heritage across various mediums, renowned for their complex structures which often feature rigorous composition, fluid linearity, and sophisticated symmetry. However, these very characteristics—including intricate interlacements and dense repetitions—present significant challenges for automated image processing and analysis, such as contour adhesion and detail loss under conditions of degradation. Therefore, this study selects representative motifs as rigorous test cases to validate the proposed parametric modeling and topological application methodology.

### 2.2. Core Algorithms and Theoretical Basis

#### 2.2.1. Dual-Channel Detection Algorithm

Traditional patterns often display morphological features such as curling, swirling, and winding. These patterns are typically nested and interlaced, resulting in highly intricate overall structures. Traditional processing methods, such as basic edge detection and threshold segmentation, struggle to accurately distinguish between the main patterns and decorative details. This difficulty is further exacerbated by material corrosion, light and shadow interference, or structural overlap within the image, which can easily lead to contour breakage or information loss [12].

**Figure 1:**
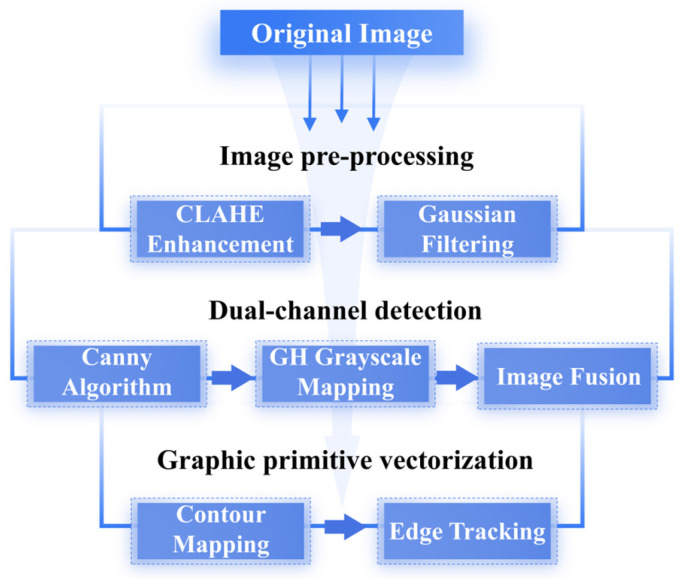
Dual-channel detection algorithm flow.

To effectively extract the main contours of traditional patterns, an edge–grayscale dual-channel synergistic method is proposed, and combines image enhancement and contour-fitting vectorization. The main steps are shown in Figure 1 and detailed as follows.

1. Image pre-processing: the original image is cropped to retain only the main part of the pattern. This part is processed in Python using the CLAHE enhancement algorithm for grayscale conversion, which strengthens the grayscale distribution contrast between the pattern and the background. Gaussian filtering is applied for smoothing and noise reduction, providing a clear boundary base for subsequent edge detection.
2. Dual-channel detection: the Canny algorithm is used for edge extraction, and morphological closure operations are applied to connect broken edges, thus improving contour continuity and reducing fragmentation. In Grasshopper, grayscale mapping converts the image into a gradient map, generating a data stream from white to black. The dual-channel data are then fused to produce a high-definition image region.
3. Graphic primitive vectorization: using contour mapping and edge-tracking methods, pixel-level contours in the image are transformed into editable vector curves. These curves are further combined with control point manipulation to realize deformation adjustment and structural extension.

#### 2.2.2. Definition of Graphic Primitives and Topological Configuration

A graphic primitive, also referred to as a pattern primitive, is the basic visual unit that constitutes pattern graphics and represents the smallest constituent element of pattern form. In the structural analysis of traditional decorative patterns, the extraction of graphic primitives facilitates the identification of stylistic features and cultural semantics, serving as a prerequisite for parametric modeling and regeneration. Graphic primitives possess independent geometric characteristics, semantic directionality, and combinability [13,14].

From the perspective of geometric features, graphic primitives have unique characteristics. Whether simple points, lines, or surfaces, or more complex geometric combinations, each primitive exhibits a distinctive geometric style that differentiates it from others. Semantic directionality refers to the fact that each primitive often carries specific cultural connotations and symbolic meanings, acting as a carrier of cultural and emotional information. Combinability indicates that, through different combination rules and methods, a limited set of graphic primitives can generate rich and varied patterns. Operations such as arrangement, splicing, repetition, and deformation allow these primitives to produce new visual effects and pattern styles.

**Figure 2:**
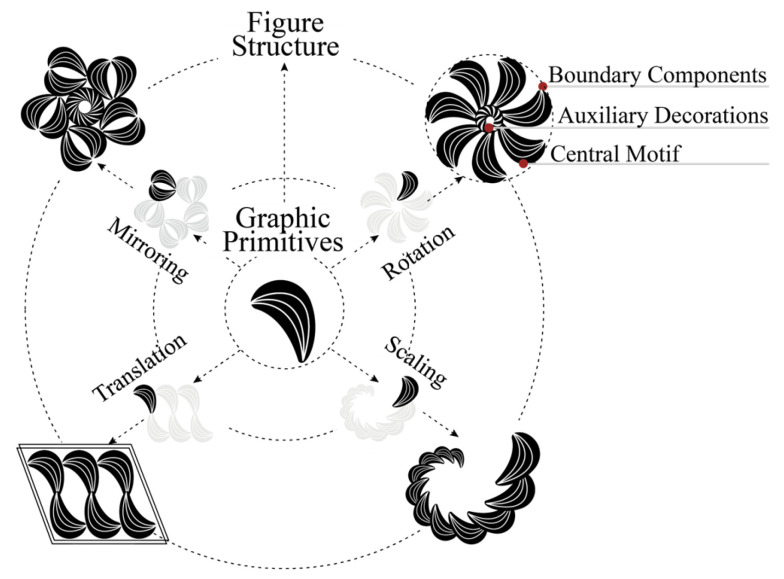
Graphic primitive and topological configuration.

Topological configuration refers to the complete compositional system of traditional decorative patterns at the visual level, representing the structured arrangement formed by combining primitives according to geometric logic. It is a composite graphic system composed of standardized primitives that follow strict spatial transformation rules, including translation, rotation, mirroring, and scaling. A typical typological structure consists of core pattern units, auxiliary decorative graphic primitives, and boundary constraint components. In digital design applications, analyzing topological configuration enables the conversion of traditional patterns from visual forms into computable models. The relationship between graphic primitives and topological configuration is illustrated in Figure 2.

#### 2.2.3. Topology Reconstruction

Topology, originally a branch of mathematics studying the invariant properties of continuity and adjacency between points in space, has evolved into a universal formal modeling framework that abstracts objects into graph structures composed of nodes and edges [15]. In pattern modeling, topological reconstruction refers to the abstraction of combinatorial relationships within a topological configuration into a graph, where nodes represent topological configurations and edges denote their connection methods. These abstract structures are then transformed into visual graphic units through geometric mapping. Topological structure modeling emphasizes the invariance of connectivity, allowing the appropriate deformation, adjustment, and reconstruction of geometric forms while maintaining invariant topological relationships (e.g., adjacency, butt joint, and containment) between topological configurations [16].

The topological structure can be represented as a graph G=(V,E), where $V=\{v_{1},v_{2},\ldots ,v_{i}\}$ denotes the set of topological configurations, with each node $v_{i}$ corresponding to a specific configuration. Furthermore, E represents the set of connectivity relationships between topological configurations, such as linear arrangements and encircling combinations.

(1) $$M:\left(G_{i},P_{i}\right)\to R^{2},$$

where M is the mapping function that transforms the topological structure into concrete geometric forms. In this process, $G_{i}$ represents the i-th topological configuration, and $P_{i}=(x_{i},y_{i},\theta_{i},s_{i})$ denotes the transformation parameters of this configuration in 2D space, including position, rotation angle, and scaling factor. Moreover, $R^{2}$ represents the 2D real number space, indicating that the mapping produces concrete graphics on a 2D plane.

Within the framework of topological structure, five types of typical topological combinations are proposed for traditional patterns: continuous arrangement, central wrap-around, symmetric splicing, nested layering, and grid inlay. By modeling the combination relationships of graphic primitives as topological graph models and defining their splicing and variation rules, patterns can achieve diversified formal evolution while preserving cultural semantics and compositional logic.

### 2.3. The Proposed Framework

**Figure 3:**
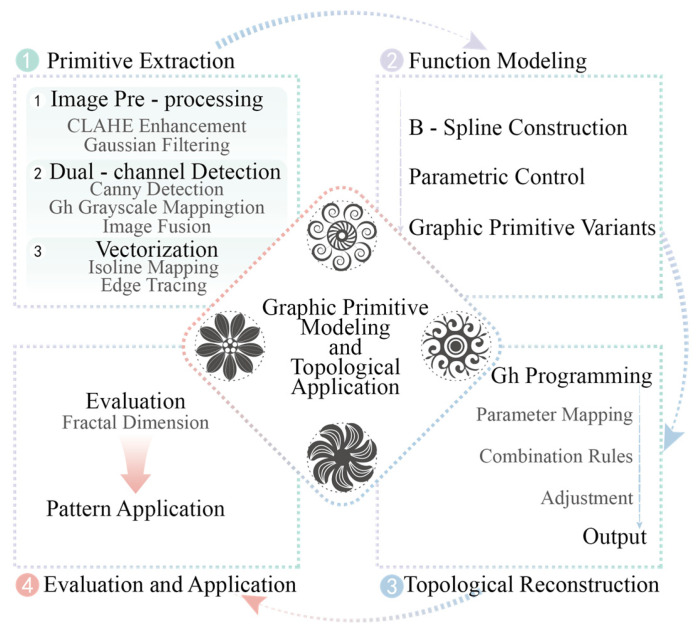
Specific path.

The pattern derivation process consists of four stages: primitive extraction, function modeling, topological reconstruction, and evaluation/application. Collected pattern images undergo preprocessing using CLAHE enhancement and Gaussian filtering. Dual-channel detection is then performed, combining Canny edge detection and Grasshopper (GH) grayscale mapping. The extracted graphic primitives are vectorized to establish parametric structures. Using B-splines as the core, functional modeling of primitives is implemented, and primitive variants are generated through parametric control. On the Grasshopper platform, topological configurations are produced by integrating geometric parameter mapping, topological combination rules, and geometric form adjustments. Finally, the fractal dimension of the generated topological reconstructions is evaluated, and the results are applied in design practice. The complete modeling path is illustrated in Figure 3 and subsequently detailed.

1. Primitive extraction: original pattern images are preprocessed using CLAHE enhancement and Gaussian filtering to improve contrast and edge clarity. Dual-channel detection, combining Canny edge detection with Grasshopper grayscale mapping, enables high-precision contour extraction of graphic primitives. Contour mapping and edge-tracking methods are then used to vectorize the primitives, establishing parametric-structured primitive models.
2. Function modeling: extracted primitives are mathematically represented as B-spline functions. Controllable deformation is achieved by adjusting parameters such as control-point displacement and curvature density, producing primitive variants through parametric control.
3. Topological reconstruction: within the topological modeling framework, Grasshopper maps geometric parameters and applies five canonical primitive combination rules: continuous arrangement, central surround, symmetric splicing, nested stacking, and grid inlay. Geometric adjustments generate diverse pattern structures, enabling systematic pattern evolution.
4. Evaluation and application: fractal dimension analysis quantifies the complexity of the generated patterns, supporting scenario-specific adaptation. Design applications, such as cultural packaging and product decoration, validate the feasibility and practicality of the proposed method.

## 3. Results

*Wadang*, a ceramic architectural component used for eaves termination in ancient structures, serves both functional and decorative purposes. The patterns adorning these components represent notable exemplars of traditional ornamentation. Characterized by rigorous composition, fluid linearity, and frequent use of central symmetry or continuous extension, these patterns were selected as test cases to validate the proposed parametric modeling and topological application methodology for traditional pattern analysis and evolution.

### 3.1. Primitive Extraction

In the structural analysis of *Wadang* patterns, graphic primitives were used to deconstruct both the formal compositional rules and cultural semantics of the motifs, forming the foundation for parametric modeling. Drawing from archeological *Wadang* specimens and documentation, this study systematically categorized the patterns in *Wadang* into four core graphic primitive types, whose morphological characteristics are detailed in Table 1.

The Han Dynasty botanical motif *Wadang* specimens feature a dominant curled structure with nested and overlapping primitives. Surface weathering has caused approximately 8% localized corrosion, resulting in contour adhesion and detail loss when using traditional threshold segmentation methods. The proposed dual-channel detection method extracted the main primitive through three stages: image preprocessing, dual-channel detection, and parameterized output.

**Figure 4:**
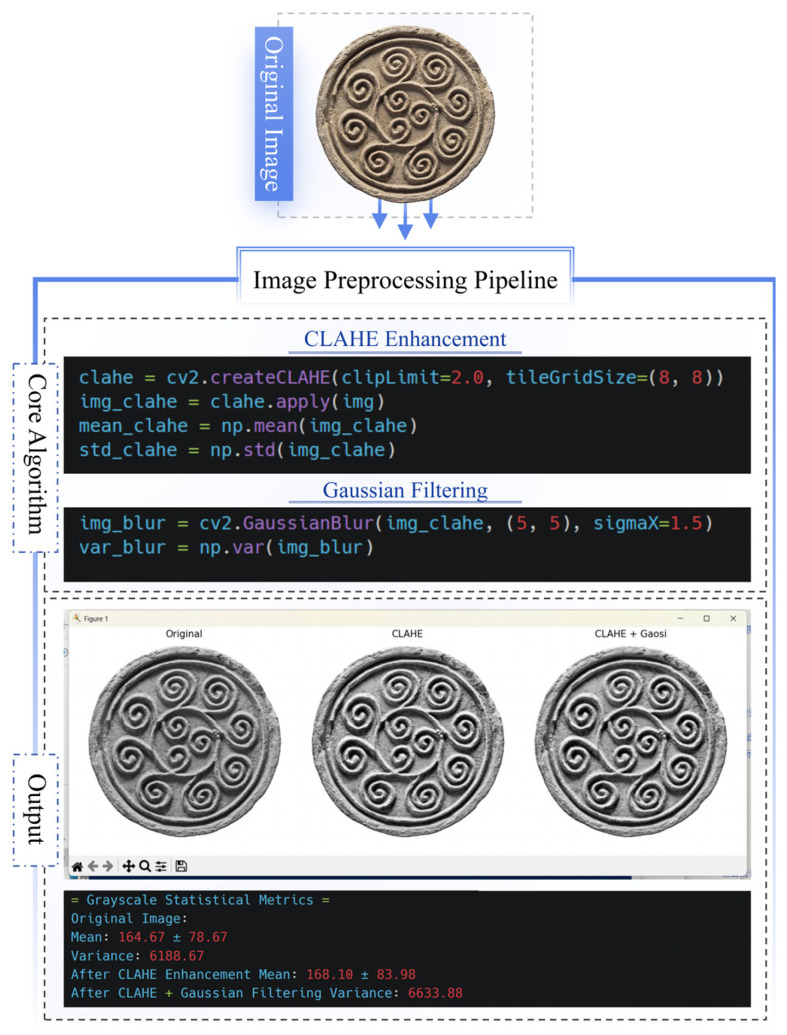
Output results of image preprocessing stage.

During image preprocessing, the CLAHE enhancement algorithm (8 × 8 grid partitions, contrast limit 2.0) was applied in Python to improve grayscale contrast, raising the average grayscale value of the core pattern region from 164.67 ± 78.67 to 168.10 ± 83.98. Subsequent Gaussian filtering (*σ* = 1.5 pixels) further clarified structural boundaries while increasing the overall image variance to 6633.88. This processed output provided an optimized foundation for primitive edge detection, with the results visually documented in Figure 4.

**Figure 5:**
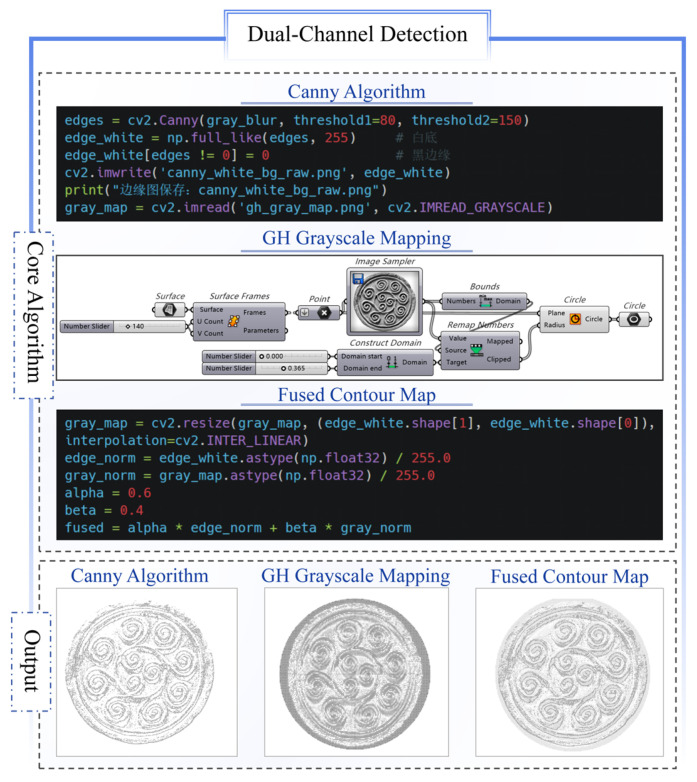
Output results of dual-channel detection stage.

In the dual-channel detection stage, the edge channel employed the Canny algorithm (dual thresholds: 80/150) to extract initial contours from the CLAHE-enhanced and Gaussian-filtered images, with the results rendered on a white background to produce standardized vector reference maps. Simultaneously, the grayscale channel generated normalized 0–1 gradient data streams using Grasshopper, mapping luminance density to capture the morphological trends and spatial features of the pattern region. Python-based linear superposition fused both channels using weighting coefficients of 0.6 (edge) and 0.4 (grayscale), producing high-contrast contour maps, as demonstrated in Figure 5.

**Figure 6:**
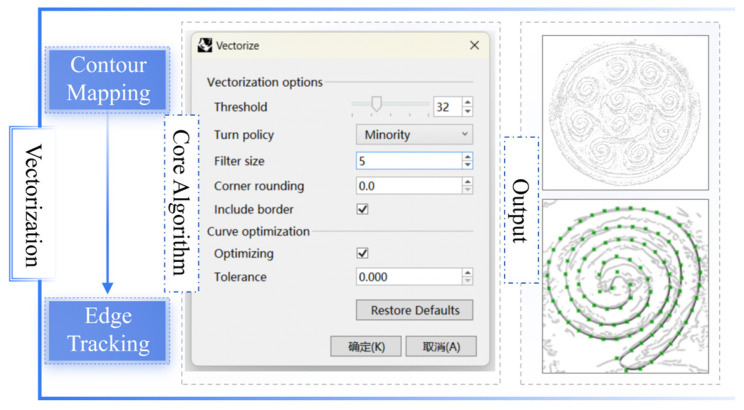
Output results of the vectorization stage of the graphic primitive.

In the graphic primitive vectorization stage, the fused images were vectorized in Rhino using the Vectorize plugin, employing contour mapping and edge-tracking methods. Using binarized pattern images as input, this process automatically extracted the main primitive contours and reconstructed their curves through parameterized threshold, smoothness, and minimum shape size settings. Compared to conventional point-set fitting approaches, this method offered operational simplicity, controllable precision, and direct generation of continuous closed curves. The output established an optimized foundation for subsequent parametric expression and topological combination modeling, with the results presented in Figure 6.

### 3.2. Function Modeling

This study employed cubic B-spline curves (degree = 3) as the core framework for graphic primitive function modeling. By configuring control point quantities, displacement amplitudes, and spatial arrangements, adjustable principal structural paths were constructed within the parameter space. This approach enables traditional pattern primitives to achieve flexible deformation and stylistic expansion while preserving their characteristic rhythmic and esthetic features [17]. The mathematical formulation is expressed as

(2) $$C\left(u\right)=\sum_{i=0}^{n}N_{i,3}\left(u\right)\cdot Q_{i},\;u\in \left[0,1\right],$$

where $C\left(u\right)$ is a point on the curve corresponding to the parameter u; $N_{i,3}(u)$ is the B-spline basis function of the *i*-th control point (degree = 3); n denotes the number of control points; and $Q=\{Q_{0},Q_{1},\ldots ,Q_{n}\}$ is the set of control points, where each control point $Q_{i}$ has coordinates represented by a number of design parameters, including width (*w*), the height (*h*), curvature density (ρ), number of segments (*n*), node vectors (*U*), and angle offset $\theta_{i}$. The control point set $Q=\{Q_{i}(x_{i},y_{i})\}$ can be expressed as a function of these parameters.

(3) $$Q_{i}=f\left(w,h,\rho ,\theta_{i}\right).$$

The graphic primitives extracted via the dual-channel detection algorithm were mathematically represented as B-spline curve functions within the Grasshopper platform. This parametric modeling process began by establishing the number and distribution of control points, followed by sequential adjustment of key variables, including displacement amplitude, point density, and angular orientation.

In the Grasshopper environment, the control point set was invoked, and the control points of the original curve were extracted through the Control Points $Q_{i}$ component to construct a normalized parameter sequence *S* = {*s*(0), *s*(1), …, *s*(*n*)}, where

(4) $$s_{i}=\frac{i}{n},i=0,\;1,\;\ldots ,\;n.$$

The parameter sequence was mapped to the Graph Mapper component to generate offset intensity values. The offset direction vector ($\vec{v}$) was defined while synchronously translating the graphic primitive’s center of gravity. This process produced an updated set of control points, which were then reconstructed into deformed curves using the Nurbs Curve component.

**Figure 7:**
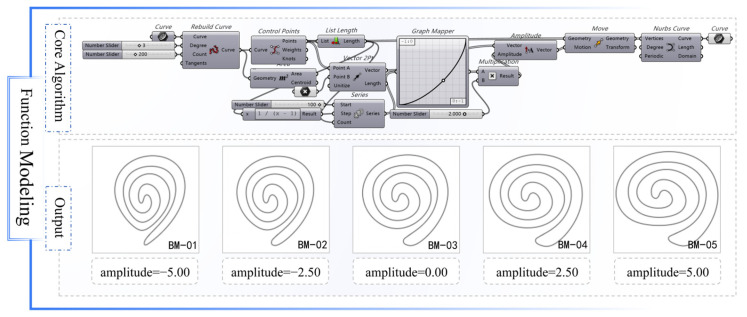
Output results of graph function modeling.

Variants with different amplitude parameter settings are illustrated in Figure 7 (using the BM-type as an example). The results demonstrate that, during the adjustment of the control point amplitude parameter from −5.00 to +5.00, the morphology of the graphic primitive underwent continuous evolution from contraction to expansion, with contour tension and curvature changes exhibiting controllable linear responses. Transitions between curve nodes remained smooth, the overall rhythmic configuration was preserved, and the model maintained high morphological consistency and cultural recognizability. The generated graphic primitives exhibited structural adaptability and could be embedded into subsequent topological structures as compositional units.

**Figure 8:**
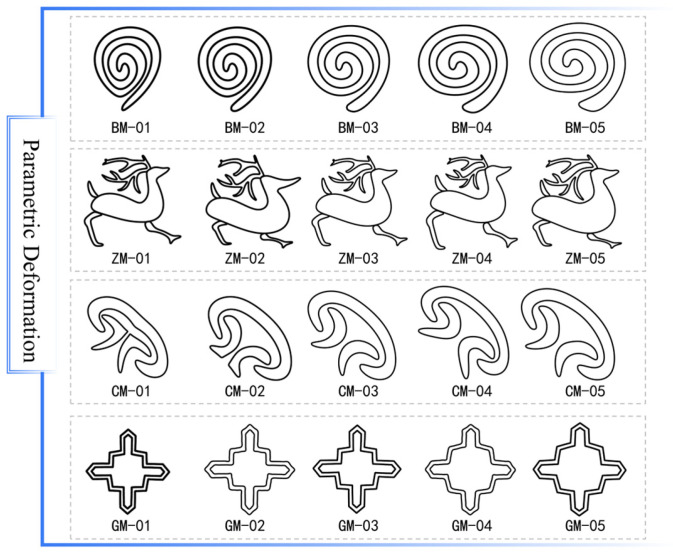
Parametric deformation results of four representative primitive categories.

Parametric deformation experiments on four representative *Wadang* primitive categories confirmed the flexibility of the modeling methodology. Figure 8 shows the resulting morphological variations, where each category maintains its core rhythmic and esthetic characteristics throughout a continuous stylistic evolution. This result validates the method’s success in preserving intrinsic design semantics during geometric transformation.

### 3.3. Topology Reconstruction

**Figure 9:**
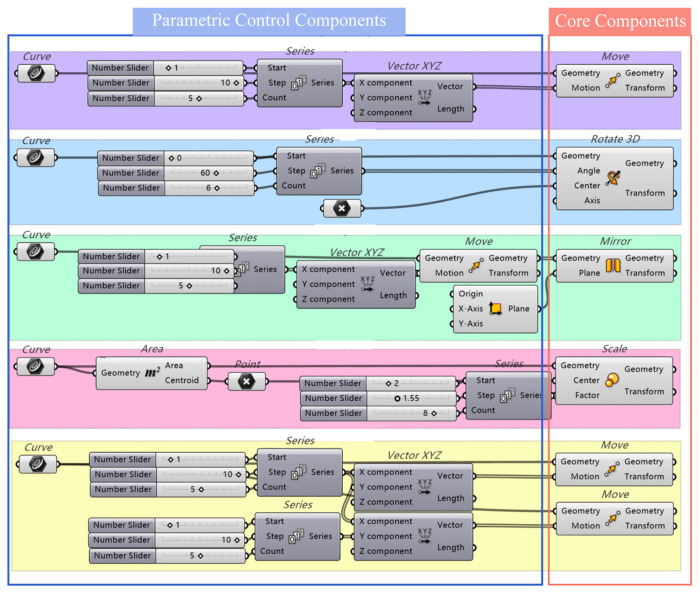
Topological reconfiguration of the battery connection diagram.

Based on the Grasshopper visual programming platform, parametric implementation frameworks for five fundamental topological connection structures were established. By defining base geometric configurations integrated with core components (Move, Rotate, Scale, Mirror), the system implemented continuous arrangement (Linear Sequence), polar coordinate-based surrounding (Radial Array), symmetric splicing (Symmetric Assembly), nested layering (Nested Stacking), and grid-embedded patterns (Modular Grid). Adjustable geometric relationships were constructed through parametric control components, with the implementation steps shown in Figure 9, allowing structural adjustments or additional units to be applied according to specific engineering requirements.

The functional modeling outputs of BM-04, ZM-03, CM-03, and GM-04 were topologically reconstructed as detailed in Table 2, with their parametric configurations systematically cataloged. The reconstructed topological constructs exhibited formal diversity while preserving cultural-semantic invariants and generative compositional rhythms.

The experimental results demonstrate that graphic primitives could be stably spliced under topological structure control, exhibiting both smooth node connections and strong consistency in both graph structure and stylistic coordination. Radial Array combinations, in particular, produced high-density radiation effects, making them well-suited for decorative applications such as gift packaging and clothing trim. Linear Sequence and Modular Grid configurations displayed enhanced regularity, effectively fulfilling design requirements for bipartite continuous patterns in home textiles and architectural materials.

### 3.4. Evaluation Application

To systematically assess the morphological complexity and structural diversity of parametrically modeled *Wadang* pattern graphic primitives, a fractal dimension evaluation method incorporating morphological characteristics was employed. This method quantitatively analyzes topologically derived graph structures using the box-counting algorithm: the contour image is embedded in multi-scale grids, and its 2D planar space-filling capacity and geometric complexity are determined by counting the number of non-empty subgrids covered by the image structure across varying grid sizes [18]. The fractal dimension *D* is computed as

(5) $$D=\underset{\epsilon \to 0}{\lim}\frac{\log N\left(\epsilon \right)}{\log(1/\epsilon )},$$

where N(ϵ) denotes the number of grids containing the pattern structure under grid side length ϵ. A higher fractal dimension *D* indicates greater detail density and geometric complexity in the image. This metric effectively quantifies morphological characteristics such as the “winding,” “branching,” and “radiating” features inherent in traditional *Wadang* patterns. Experimental thresholds indicate that *D* > 1.5 corresponds to intricate configurations suitable for premium gift decoration, whereas *D* < 1.2 aligns with minimalist esthetics ideal for modern interior design contexts.

**Figure 10:**
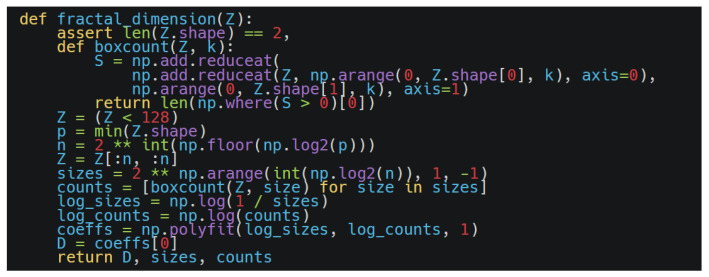
Fractal dimension core code.

Five generated topologically derived configurations were processed using a Python-based fractal dimension evaluation framework. The core algorithm workflow, as illustrated in Figure 10, was applied to quantify the structural complexity of each configuration.

The analysis of the *D*-values indicates that different topological combinations significantly influenced pattern complexity: the mesh tessellation (Modular Grid) and continuous arrangement (Linear Sequence) configurations exhibited higher *D*-values, indicating more intricate and diverse structural characteristics, as shown in Table 3.

**Figure 11:**
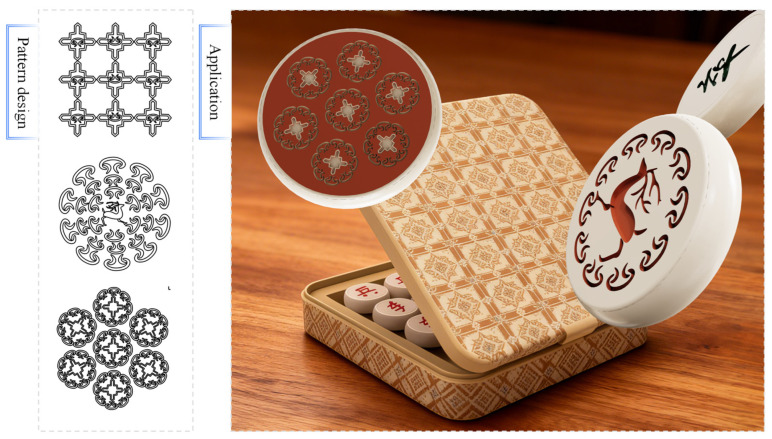
Application of the generated patterns in design.

The proposed parametric design method, which enables rapid adjustment of morphological parameters and topological combinations for easy iteration, is well-suited for modern cultural product design—specifically chess, for decorating pieces and boards. In practice, abstracted traditional graphic primitives (e.g., simplified *Wadang* motifs) were applied to key chess components: patterns on rooks, cloud-derived motifs on bishops, and geometric combinations on board borders. Using moderate saturation in classic chess hues (black, red) to avoid distracting from gameplay, the patterns balance traditional cultural connotation with modern minimalism; as shown in Figure 11, they enhance the chess set’s visual identity while maintaining playability.

## 4. Discussion

### 4.1. Robustness Evaluation Under Degraded Conditions

To further evaluate the robustness of the proposed dual-channel image processing pipeline, additional experiments were conducted under artificially degraded conditions. Two common types of image noise—Gaussian noise and salt-and-pepper noise—were added to the input images to simulate typical deterioration scenarios such as surface corrosion, aging, and low-quality scanning.

Gaussian noise was generated with a mean of 0 and a standard deviation of 25, while salt-and-pepper noise was applied with a noise density of 0.02, replacing 2% of pixels with maximum (255) or minimum (0) intensity values. These degraded images were then processed using the proposed method to assess its ability to accurately extract and reconstruct pattern primitives under adverse conditions.

**Figure 12:**
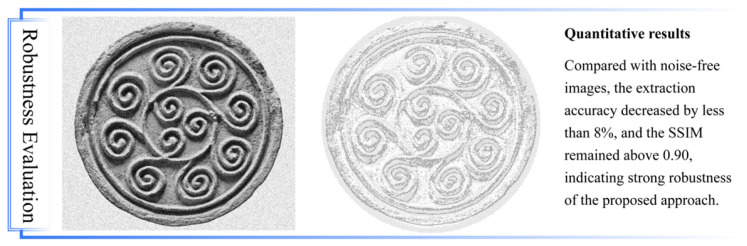
Robustness evaluation.

The proposed pipeline maintained stable performance even under degraded image conditions. As shown in Figure 12, both Gaussian noise and salt-and-pepper noise introduced noticeable distortions to the original images. However, the dual-channel detection process successfully extracted continuous contour information and reconstructed graphic primitives with minimal accuracy loss.

### 4.2. Qualitative Comparison with Existing Methods

**Figure 13:**
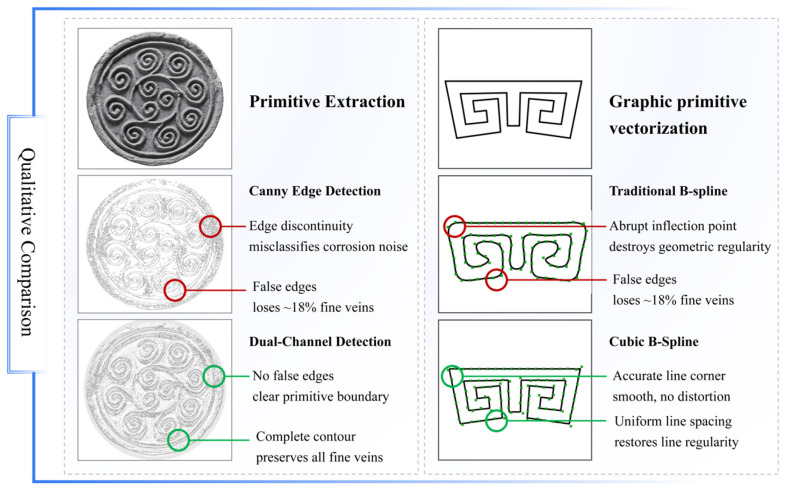
Comparison of primitive extraction and curve modeling.

To intuitively validate the advantages of the proposed dual-channel detection and cubic B-spline modeling, two representative conventional methods were selected for qualitative comparison: classic Canny edge detection (for primitive extraction) and traditional B-spline modeling (for curve fitting). The qualitative analysis focuses on three key dimensions: contour integrity, esthetic fidelity, and cultural information preservation. The results are presented in Figure 13.

The qualitative comparison of primitive extraction and curve modeling confirms that the proposed framework outperforms conventional methods in addressing the core challenges of historical *Wadang* digitalization. For primitive extraction, the dual-channel detection effectively resolves the limitations of Canny edge detection, preserving complete contour details critical for cultural information retention. For curve modeling, the cubic B-spline method overcomes the esthetic distortion of traditional B-splines: it avoids abrupt inflection points and uneven grid spacing in geometric Huiwen *Wadang* deformation, maintaining the “rigid regularity” that defines the esthetic of traditional geometric motifs.

### 4.3. Experimental Results and Discussion

The experimental results confirm the core hypothesis that a structured image processing pipeline is paramount for robust analysis of traditional patterns. The dual-channel (edge-grayscale) detection algorithm proved critical in overcoming the limitations of conventional single-channel methods (e.g., basic Canny or thresholding), effectively mitigating contour adhesion and detail loss in degraded historical images. This underscores the value of multi-source feature fusion for complex heritage imagery.

The subsequent parametric B-spline modeling and topological reconstruction steps successfully translated the extracted visual data into a flexible, rule-based system. This transition from raster-based image analysis to vector-based geometric modeling is the key innovation, enabling both the preservation of original stylistic rhythms and the generation of novel, structurally coherent patterns. The fractal dimension analysis provided an objective, quantifiable metric to evaluate the visual complexity of these outputs, linking algorithmic parameters to perceptual outcomes.

The broader implication is a demonstrated pipeline for converting archival images into editable, semantically structured digital assets. This has immediate applications in cultural heritage informatics for creating scalable vector databases, and in creative industries for streamlining design workflows. The method’s reliance on open-source tools (Python, Grasshopper) enhances its reproducibility and potential for extension.

## 5. Conclusions

This study has proposed a novel pattern regeneration design method based on graphic primitives and topological reconstruction. By integrating mathematical logic-driven design into traditional pattern regeneration, this research effectively addresses the deficiencies in structural analysis and form generation mechanisms as identified in the existing literature.

The core of the proposed framework involves a dual-channel detection algorithm for robust primitive extraction, B-spline curves for unified functional representation and parametric control, and a topological reconstruction path to generate diverse configurations through combinatorial variations. The application of fractal dimension analysis provides a quantitative metric for evaluating pattern complexity, validating the method’s adaptability and ensuring its engineering applicability for managing morphological complexity and stylistic evolution.

This framework bridges computational design with cultural heritage preservation, offering significant potential for application in cultural creative industries. Despite its effectiveness, the proposed method has certain limitations. The dual-channel detection algorithm may still struggle with highly degraded or low-contrast images. Additionally, the current topological rules are limited to 2D structures, which may not fully capture the spatial complexity of some traditional patterns. Future work will focus on extending the topological rules to three-dimensional surfaces and integrating machine learning techniques to enhance the automation and adaptability of the pipeline for a broader range of cultural motifs.

## Figures and Tables

**Table 1:** The classification of graphic primitives in major *Wadang* patterns.

Type	Botanical Motif	Zoomorphic Motif	Cloud Motif	Geometric Motif
Image	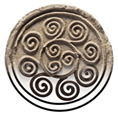	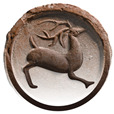	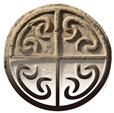	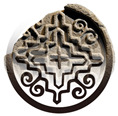
Primitive	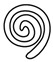	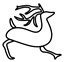	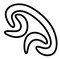	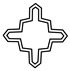
Features	Axial symmetry	Dynamic contour	Fluid curvature	Regular polygon
Label	BM-	ZM-	CM-	GM-

**Table 2:** Topological reconstruction and parametric configurations of selected motifs.

Topology Types	Linear Sequence	Radial Array	Symmetric Assembly	Nested Stacking	Modular Grid
Topo-Configuration	Main axis layout, Linear rhythm	Circular layout, Radiating structure	Mirror design, Splice layout	Nested layers, Hierarchical order	Matrix arrangement, 3D effect
Topo-Relations *	$G_{i}↔G_{i+1}$	$G_{i}\in R(G_{c})$	$G_{j}=R_{mirror}(G_{i})$	$G_{i+1}\subset G_{i}$	$G_{i,j}\in Grid(m\times n)$
Topo-Parameters	a. Count b. Spacing c. Angle	a. Radius b. Rings c. Angle step	a. Axis position b. Orientation c. Direction	a. Depth b. Scale factor c. Density	a. Grid b. Edge length c. Element scale
Topo-Graph	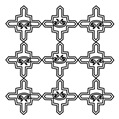	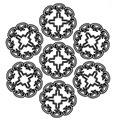	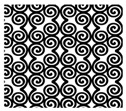	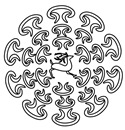	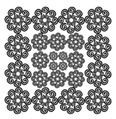

* $G_{i}$, $G_{i}$: input primitives; R: radiation range; $G_{c}$: composite pattern; ↔: bidirectional parametric dependency; ∈: membership relationship; ⊂: inclusion relationship.

**Table 3:** The D-values of topological reconstruction.

Type	Linear Sequence	Radial Array	Symmetric Assembly	Nested Stacking	Modular Grid
*D*-value	1.7766	1.8641	1.8473	1.8361	1.8760

## Data Availability

No new data were generated or analyzed in this study. The findings are based on theoretical methodology development and synthesis of the existing literature, as cited in the references.

## Abbreviations

The following abbreviations are used in this manuscript:

B-spline	Basis-spline
CLAHE	Contrast-Limited Adaptive Histogram Equalization
GH	Grasshopper
